# Focal mechanical vibration motor effects on the finger tapping in healthy volunteers

**DOI:** 10.3389/fspor.2025.1674876

**Published:** 2026-01-20

**Authors:** Tommaso Di Libero, Angelo Rodio, Chiara Carissimo, Gianni Cerro, Cecilia Provenzale, Annalisa D’Ermo, Guido Maria Filippi, Vito Enrico Pettorossi, Luigi Ferrigno, Enrico Marchetti, Luigi Fattorini

**Affiliations:** 1 Sustainable Living Concept Xlab Marco Marchetti, Department of Human Sciences, Society and Health, University of Cassino and Southern Lazio, Cassino, FR, Italy; 2 Department of Medicine and Health Sciences “Vincenzo Tiberio,” University of Molise, Campobasso, Italy; 3 Department of Electrical and Information Engineering, University of Cassino and Southern Lazio, Cassino, Italy; 4 Department of Neuroscience, School of Medicine, Faculty of Medicine and Surgery, Università Cattolica del Sacro Cuore, Rome, Italy; 5 Department of Medicine and Surgery, Human Physiology Section, Università degli Studi di Perugia, Perugia, Italy; 6 Department of Occupational Medicine, Epidemiology and Hygiene, Istituto Nazionale Assicurazione contro gli Infortuni sul Lavoro (INAIL), Rome, Italy; 7 Department of Physiology and Pharmacology “Vittorio Erspamer,” Sapienza University of Rome, Rome, Italy

**Keywords:** coordinative abilities, mechanical stimulation, motor performance, neuromotor plasticity, proprioception, wearable devices

## Abstract

**Introduction:**

Focal mechanical vibration is a therapeutic intervention based on oscillatory mechanical vibrations applied to specific anatomical structures. These vibrations can be generated by portable devices and transmitted to targeted muscles, joints, or tissues. Focal vibration stimulates local receptors, producing a sensory input flow that induces neural reorganization with functional effects such as improved motor performance, enhanced sensory perception, and reduced pain or discomfort. Several studies have investigated the effects of proprioceptive training (PT), particularly in improving strength and muscular endurance. Other research has reported changes in the rate of strength development. While these aspects have been well explored, neuromotor coordination abilities, which refer to the ability to organize and perform complex motor tasks effectively and efficiently, remain less well examined. In fact, this study aimed to fill this gap by examining the effects of PT on coordinative abilities, specifically through the finger tapping task.

**Methods:**

The study involved college students who were divided into a control group and an experimental group. The assessed parameters included tapping number, movement displacement, and intertapping interval (i.e., tapping frequency).

**Results:**

The results showed increases in endurance time and the number of taps only in the treated group, although a slight increase was also observed in the control group. These effects were recognized both acutely, lasting up to 24 h, and chronically, persisting for up to 4 weeks.

**Discussion:**

The study contributes to the existing literature on vibration interventions and enhances our understanding of the role of focal vibration in improving motor performance.

## Graphical Abstract

**Figure:**
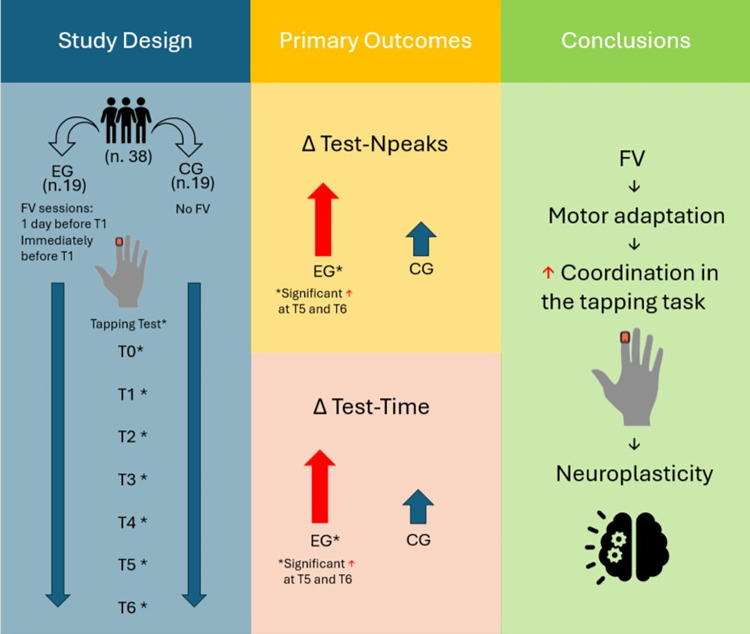

## 1 Introduction

Previous research has established that adequate proprioceptive training (PT) (1) can improve motor execution. Among the various methods, an effective protocol for administering focal vibration (FV), capable of powerfully stimulating muscle spindles, has been described and reviewed as a valid proprioceptive training approach by Filippi et al. (2) and Fattorini et al. (3–5). These studies identified optimal vibratory parameters that elicit positive, significant, and long-term persisting after-effects on motor performances, including strength, power, rate of force development, and fatiguability. Conducted in both healthy and diseased individuals, these studies showed positive and long-lasting motor improvements even in the absence of concomitant physical training. Such improvements have been observed in both simple tasks, such as leg flexion-extension (6), and more complex motor tasks involving multiple joints (7, 8), suggesting a reduction in metabolic involvement at comparable external workloads following FV. This effect has been interpreted as resulting from improved agonist–antagonist coordination and increased muscular efficiency. Neurophysiological studies using transcranial magnetic stimulation (TMS) have shown that FV training may induce plastic changes in the primary motor cortex, characterized by more accurate inhibition of antagonist muscles (9, 10). Filippi et al. (2) hypothesized that such neural reorganization results from non-specific sensory stimulation capable of inducing long-term hetero- and homo-synaptic effects in the CNS, such as long-term potentiation (LTP). Based on several studies, the authors suggested that the observed motor improvements are likely due to enhanced neural control, supported by both increased processing of afferent spindle signals and improved integration of internal and external spatial coordinates.

Pettorossi et al. (11), in studies on balance impairment after neck muscle fatigue, reported that this intervention enhances proprioceptive sensitivity and the central processing of sensory input, thereby reinforcing spatial accuracy even after fatigue. The observed adaptations in central motor drive and increased proprioceptive sensitivity provide a possible rationale for the effects of FV described in these studies. Neurophysiological investigations (9, 10) have demonstrated a significant improvement in agonist–antagonist muscle balance. This agonist–antagonist interplay is crucial not only for reducing joint impedance but also for enhancing motor abilities, coordination, and resistance to fatigue (12–14). In repetitive movement tasks, improved agonist–antagonist activation may increase the number of repetitions, whereas in strength-based tasks, it may enhance force or power output. However, although literature suggests improvements in coordination abilities after FV, more specific and validated experimental studies are still required. Finger tapping (FT) is a simple motor task commonly used in oculo-manual coordination tests and is widely adopted to evaluate motor coordination in various clinical conditions, including Parkinson’s disease (15, 16), Alzheimer’s disease (17–20), developmental coordination disorder (21), ataxia (22), and poststroke recovery (23). Furthermore, FT and its associated neural activation patterns are used to characterize upper limb motor function (24) and to distinguish precision-demanding from simple finger movements (25). These studies have shown that activation of the primary motor cortex varies depending on the nature of the FT task. Control of finger movements is particularly demanding due to the low innervation ratio, which requires the central nervous system to coordinate a large number of motor units with high spatial resolution (26). FT is characterized by a low metabolic cost. Previous studies have shown that muscle contractile properties remain unchanged after prolonged FT tasks (27), and no significant reductions in maximal voluntary contraction force have been observed (28). In addition, voluntary muscle activation and spinal excitability remain unaltered following fast FT performed at maximal speed (26, 29–31), suggesting that FT-related fatigue is primarily of supraspinal origin. On the other hand, FT is a repetitive, alternating task that relies heavily on precise muscle coordination and an appropriate agonist–antagonist balance, which minimizes joint mechanical impedance while ensuring joint stability. A decline in cortical control of this balance increases joint impedance and internal work, which may limit task repetition (32–39). Therefore, this study aimed to investigate the effects of a proprioceptive training intervention using FV on performance during a finger tapping task performed until exhaustion. The potential of FV to enhance agonist–antagonist coordination and proprioceptive sensitivity may prolong task execution and delay the onset of central fatigue.

## 2 Materials and methods

### 2.1 Participants

A total of 38 students from the University of Cassino and Southern Lazio participated in this study, including 16 women (age, 22.9±0.88 years; height, 1.65±0.06 m; Weight, 61.9±9.62 kg) and 22 men (age, 24.2±3.04 years; height, 1.75±0.05 m; weight, 73.42±6.08 kg). Of the selected participants, 19 were randomly assigned to the experimental group (EG) and 19 to the control group (CG). Randomization was performed using a computerized random number generator (RAND function in Microsoft Excel), with group allocation conducted by an independent researcher not involved in data collection. The EG received focal vibration administration as described below and performed the FT task, while the CG performed the FT task only. All participants were informed about the study procedures and provided written consent prior to participation. This study was approved by the Institutional Review Board of the University of Cassino and Southern Lazio (no. 24777.2022.12.12) and conducted in accordance with the 1964 Declaration of Helsinki.

### 2.2 Study protocol

**Figure 1:**
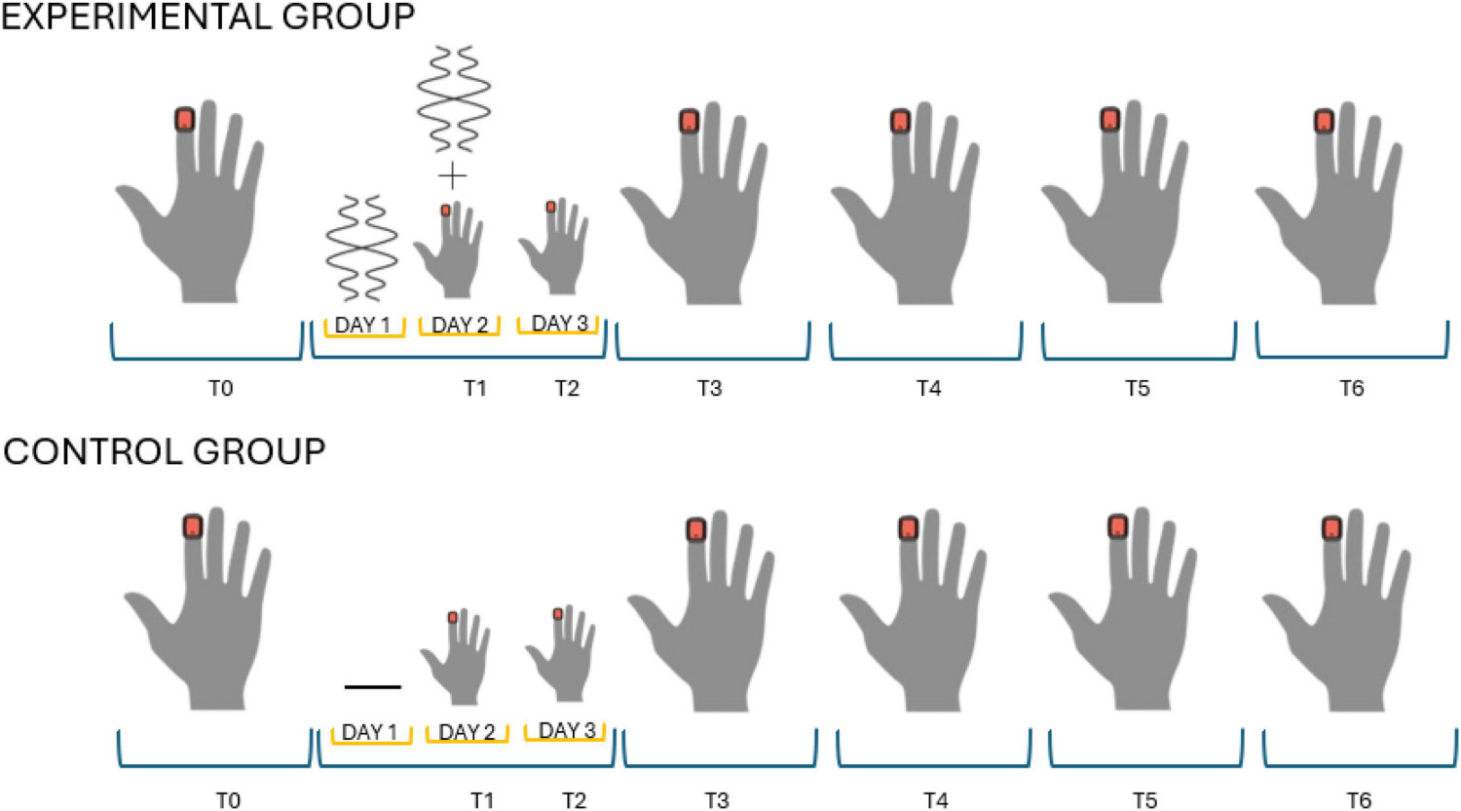
Experimental protocol for the EG and CG. T0 represents the baseline assessment. The orange square indicates the placement of the IMU sensor on the fingertip. Assessments were performed weekly, except for T1 and T2, which were conducted 1 day apart. In the EG, vibration was delivered on the day before and immediately prior to T1. The CG followed the same timeline without vibration.

Over the course of 6 weeks, both groups followed a structured protocol. During the first week, participants performed the FT task (T0), serving as the baseline assessment. In the second week, the EG received vibration treatment on day 1, followed by FT on day 2 (T1, immediately after vibration) and again on day 3 (T2). In subsequent weeks, FT assessments were conducted on days 14 (T3), 21 (T4), 28 (T5), and 35 (T6). The CG followed the same schedule but did not receive vibration treatment. All experimental phases are shown in Figure 1.

### 2.3 Intervention and measurement setup

#### 2.3.1 Finger tapping test

**Figure 2:**
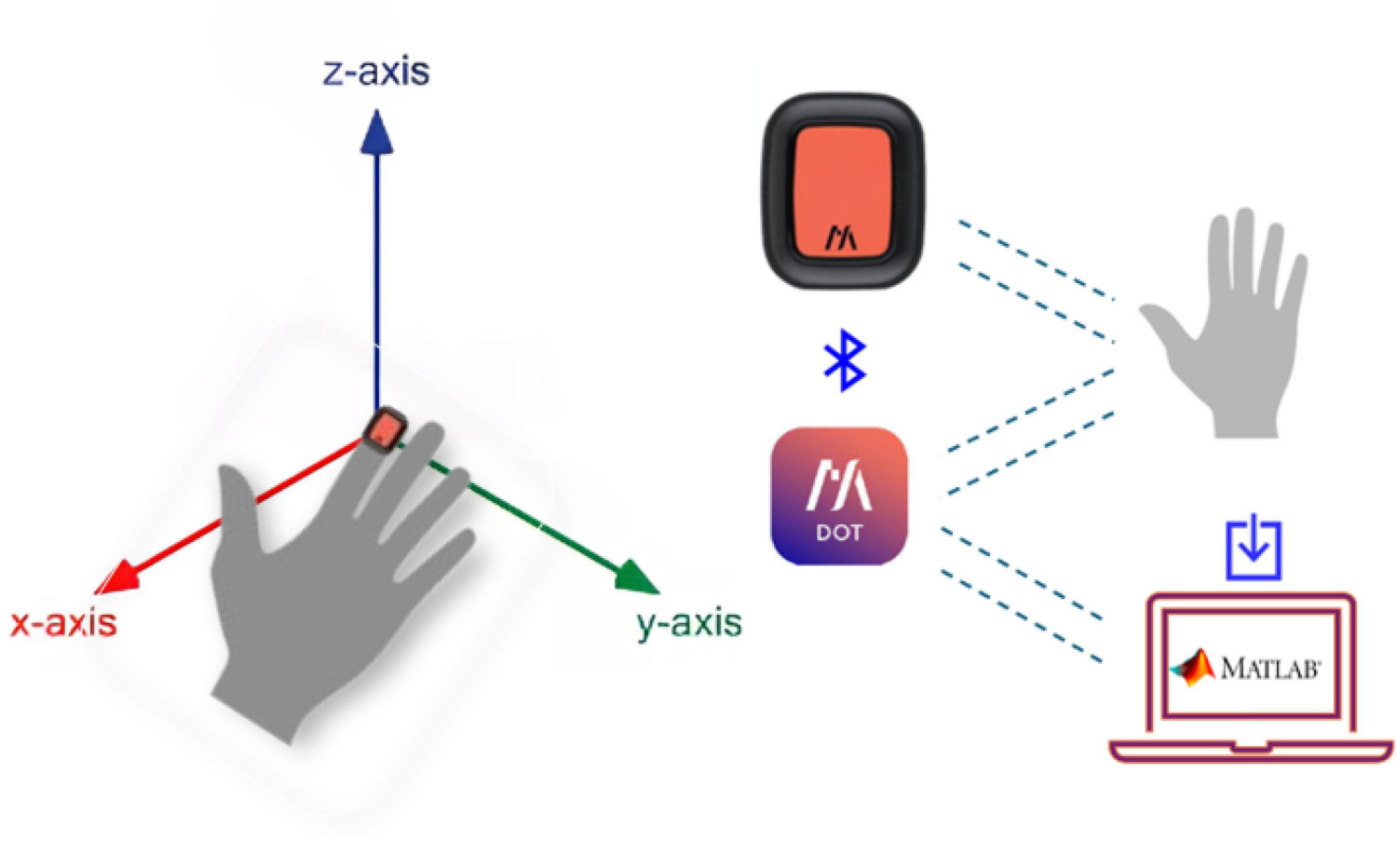
Measurement setup.

Participants were seated comfortably with the elbow flexed at approximately $90^{\circ }$, the forearm supported on a table, and the hand placed palm-down, as shown in Figure 2. The dominant hand was used, as identified by the Edinburgh Handedness Inventory (40). After a 3-s countdown, participants tapped rapidly with their index finger at a self-selected pace, using a full vertical excursion. The task was stopped voluntarily at the onset of fatigue or discomfort. Finger movements were recorded using IMU sensors. To isolate finger motion, participants were instructed to avoid moving their shoulder or elbow.

#### 2.3.2 Kinematic measurements

A wearable triaxial IMU was used to record acceleration and angular velocity at 120 Hz. The sensor was positioned on the dorsal side of the distal phalanx of the index finger and secured with elastic adhesive tape, ensuring stability without interfering with finger movement. This setup has been previously validated for use in similar motor tasks.

#### 2.3.3 Vibration intervention

The EG received focal vibration via the ${\text{CRO}}^{®}$SYSTEM device (NEMOCO srl, Italy), delivering stimulation at 100 Hz (8). Each session lasted 45 min and was divided into three 15-min blocks, with 1-min recovery intervals between blocks. Participants were seated comfortably with the forearm resting on a soft surface and the hand positioned on a support bracket connected to the device. A consistent grip was maintained under operator supervision to ensure effective delivery of the stimulus.

#### 2.3.4 Data processing and analysis

Raw IMU data were processed to extract movement characteristics. Analysis focused on vertical acceleration (*z*-axis) as a proxy for finger displacement. Four main parameters were extracted:

- total test duration (Test-time);
- total number of peaks (Test-Npeaks);
- mean interpeak time in 5-s non-overlapping windows (IntTime); and
- mean peak acceleration in 5-s non-overlapping windows (Accpeak).

**Figure 3:**
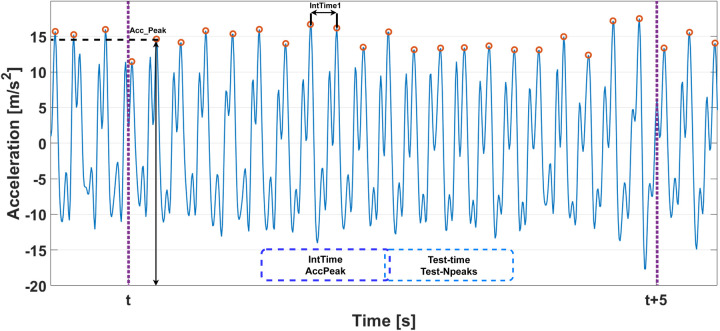
Algorithm diagram used to compute tapping movement parameters.

As shown in Figure 3, the signal was segmented into disjoint 5-s windows. Within each window, peaks and inter-peak intervals were calculated. The algorithm used for this analysis has been validated in previous studies (41–44).

## 3 Statistical analysis

For all parameters analyzed, the values from T1 to T6 were normalized by subtracting the corresponding baseline value at T0, as suggested by Jooss et al. (45). To indicate this transformation, the delta symbol (Δ) was added before each parameter name, signifying that the data represent changes relative to baseline. This approach yielded output magnitudes that retained the same physical units as the original variables and enabled evaluation of each participant’s performance relative to their individual baseline. This normalization ensured that the observed changes more accurately reflected the effects of vibration, rather than pre-existing differences in baseline tapping performance. To assess the effects of the intervention, a two-way mixed ANOVA was performed for each parameter, with *test phase* (T1–T6) as the within-subject factor and *group* (CG vs. EG) as the between-subject factor. Normality of residuals was assessed using the Shapiro–Wilk test. The assumption of sphericity was evaluated using Mauchly’s test, and when violated, the Greenhouse–Geisser correction was applied. Effect sizes for significant ANOVA results were calculated and reported as omega squared ($\omega^{2}$).

## 4 Results

**Figure 4:**
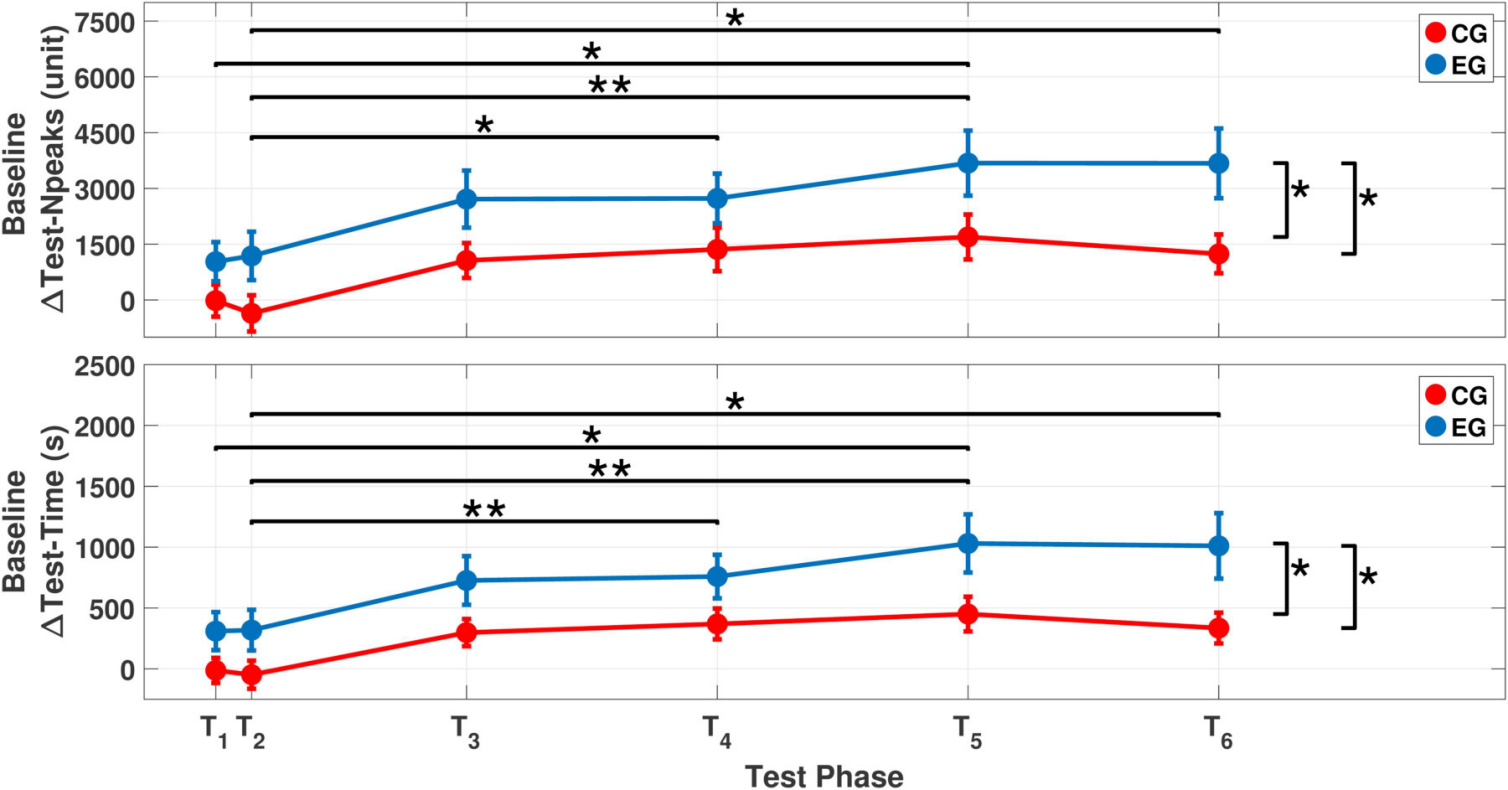
Comparison between the CG and EG for ΔTest-Npeaks (*top*) and ΔTest-Time (*bottom*). Horizontal lines represent statistical comparisons across test phases, while vertical lines indicate between-group comparisons at T5 and T6. An asterisk (*) indicates a p-value less than 0.05, while double asterisks (**) denote a p-value less than 0.01. For the test phase comparison (i.e., horizontal line), the asterisk indicating statistical significance is placed above the line.

The mixed ANOVA showed significant effects of both test phase (p<0.001) and group (p=0.009) on the Test-Npeaks parameter (see Tables 1 and 2). These main effects indicated significant differences between the CG and EG at the $T_{5}$ (p=0.05) and $T_{6}$ (p=0.021) phases (see Figure 4). *Post-hoc* analyses with Bonferroni correction highlighted significant differences between early test phases ($T_{1}$ and $T_{2}$) and later phases ($T_{1}-T_{5}$ (p=0.015); $T_{2}-T_{4}$ (p=0.014); $T_{2}-T_{5}$ (p=0.006); $T_{2}-T_{6}$ (p=0.024)) (see Figure 4). No significant interaction was observed between the test phase and group factors.

**Table 1:** Results of the mixed ANOVA (within-subject effects) showing the influence of test phase on measured parameters.

Test phase effect (within-subject effects)	p	F	df	$\omega^{2}$
Test-Time (s)	<0.001	7.374	3.474	0.088
Test-Npeaks (unit)	<0.001	7.157	3.645	0.085
IntTime (s)	0.076			
Accpeak (ms^−2^)	0.347			

Reported values are the p-values for each parameter. For statistical significant results, the table also reports the F-value, degree of freedom (df), and effect size ($\omega^{2}$). The latter indicates a medium effect size for both the Test-Time and Test-Npeaks parameters.

**Table 2:** Results of the mixed ANOVA evaluating the effect of group (between-subject effects) on parameters of interest.

Group effect (between-subject effects)	p	F	df	$\omega^{2}$
Test-Time (s)	0.006	8.545	1	0.095
Test-Npeaks (unit)	0.009	7.649	1	0.085
IntTime (s)	0.477			
Accpeak (ms^−2^)	0.709			

p-Values indicate the significance of group differences for each parameter. For statistically significant results, the table also reports the F-value, degree of freedom (df), and effect size ($\omega^{2}$). The latter indicates a medium effect size for both the Test-Time and Test-Npeaks parameters.

**Figure 5:**
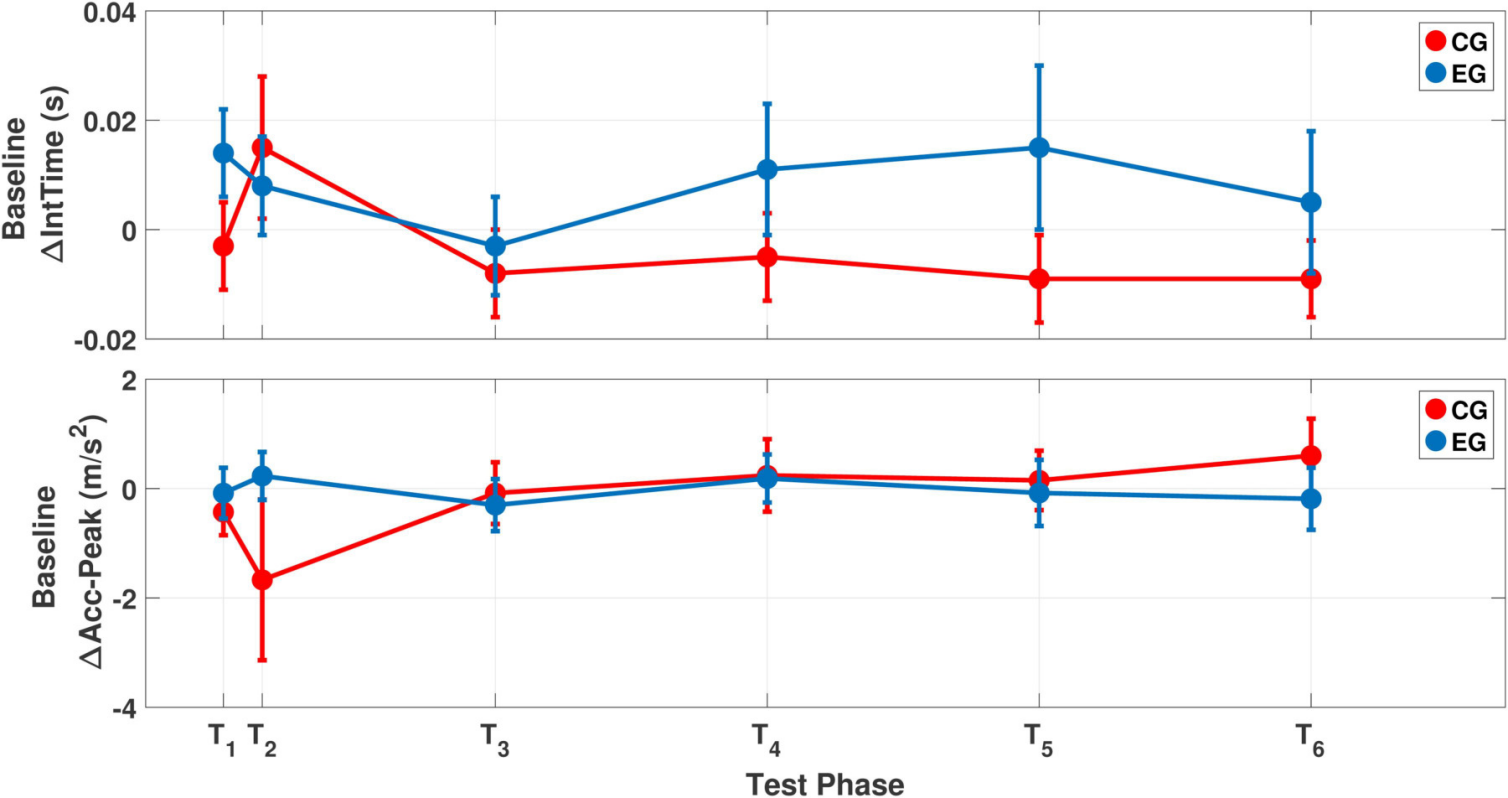
Comparison between the CG and EG for ΔIntTime (*top*) and ΔAccpeak (*bottom*).

The statistical analysis of the ΔTest-Time parameter showed statistically significant effects of both the test phase (p<0.001) and group (p=0.006) (see Tables 1 and 2). These main effects highlighted significant differences in ΔTest-Time between the two groups at test phases $T_{5}$ (p=0.031) and $T_{6}$ (p=0.021) (see 4). Bonferroni-corrected *post-hoc* test showed significant differences in test phases for the following comparisons: $T_{1}-T_{5}$ (p=0.011); $T_{2}-T_{4}$ (p=0.009); $T_{2}-T_{5}$ (p=0.005); $T_{2}-T_{6}$ (p=0.034) (see 4). The analysis did not show any statistically significant interaction between the test phase and group. No statistically significant effects of test phase or group were observed for ΔIntTime and ΔAccpeak parameters. Figure 5 shows the mean values of ΔIntTime and ΔAccpeak parameters for each test phase and group. It is possible to observe how the CG and EG paths overlap in each of the considered parameters.

## 5 Discussion

FT is a repetitive motor task that requires accurate muscle coordination based on adequate agonist–antagonist alternation of muscle activation. It is typically used to evaluate fine motor skills as well as in neuropsychological assessments, while neurological examination often includes a more qualitative evaluation of finger-to-thumb tapping. In repetitive finger movement tasks, the literature reports that fatigue is associated with increased intracortical inhibition in the primary motor cortex (M1), which suggests the presence of central rather than local fatigue (28, 29, 39, 46). This pattern indicates that fatigue development is mainly induced by changes in supraspinal motor drive (32). At the same time, several studies, including those by Filippi et al. (2) and Fattorini et al. (3–5), suggest that a specific FV stimulation protocol can elicit strong spindle activation capable of promoting plastic changes in the central nervous system. These adaptations have been hypothesized to contribute to improvements in motor performance in both healthy and clinical populations, likely by improving joint impedance through a rebalance of the agonist–antagonist muscle interplay; for more information, see Filippi et al. (2) and Fattorini et al. (4–6, 47).

The present paper aimed to evaluate the short- and long-term effects of a specific proprioceptive training using FV on coordinative abilities by assessing kinematic parameters of a simple motor task consisting of repetitive FT performed until exhaustion and by comparing the data with those of the CG. The findings reveal several interesting aspects. First, a large intra-subject variability was observed at baseline (T0) in both groups, likely due to differences in intraindividual manual skills. For this reason, all parameters were normalized to baseline values, identified through the Δ symbol, to better highlight and intercept potential changes over time (45). The most notable findings concern the effects induced by the FV intervention, which were evident as early as T1 and progressively increased over time, achieving statistical significance compared with T2 from the first week onward (T3, T4, T5, and T6 vs. T2), as illustrated in Figure 4. Importantly, within the CG, although a clear improvement in performance was noted, likely attributed to a training effect, no statistically significant changes were observed. These results highlight the role of the specific FV protocol in eliciting a significant and persistent modulation of motor performance. Based on previous literature, such after-effects are likely sustained by adaptive plastic changes in the central nervous system, as suggested by Filippi et al. (2) and Fattorini et al. (4–6, 47).

Regarding the magnitude of the observed effects, it is worth noting that Filippi (8) also reported significant improvements immediately after treatment and 1 week later in a Wingate task involving the lower limbs. However, the performance improvements observed in that study were relatively smaller compared to the present study. In the current investigation, the task was specifically selected to emphasize the spatiotemporal effects of FV on movement control by using a repetitive movement that requires the coordination of highly specialized muscles involved in finger tapping. These muscles possess a high receptor density for perceiving and modulating tensional states, along with a low innervation ratio, both of which are crucial for precise motor execution. It is plausible that this high receptor density enables a high-intensity response in the spindle neural afferent to the central nervous system during FV. Such increased intensity might justify the after-effect amplitude. In light of this, the central nervous system adaptations hypothesized in prior studies (5, 8) may offer a possible framework for interpreting the observed effects.

Although differences between groups can be observed across all time points in Figure 4, these differences become more pronounced and statistically significant at T5 and T6. This is likely related to the high variability in the results and may reflect individual differences in manual ability related to fine motor tasks performed in daily life (45). Shifting the attention to movement kinetic parameters (see Figure 5), the marked increases in ΔIntTime and ΔTest-NPeaks were not associated with relative kinematic changes, as confirmed by statistical analyses of both between-group and within-group differences over time. This result is not unexpected, as several studies have reported that fatigue during FT is mainly of central rather than local origin (32). Indeed, it is commonly observed that individuals, particularly those trained in fields such as music, can perform repetitive finger movements for prolonged durations without alterations in movement speed or precision. In this regard, Rosenkranz et al. (48) studied professional musicians and reported enhanced motor cortical excitability attributable to long-term finger training (48).

At the same time, Marconi et al. (9, 10) reported that TMS after FV treatment showed long-lasting, beneficial cortical modulation of the agonist–antagonist muscle balance, which was associated with the improvement of motor coordination of the joint belonging to the vibrated muscle and its antagonist. These motor changes were correlated to modulation of short-interval cortical inhibition and cortical excitability, as further confirmed by Calabrò et al. (49). These findings from the literature are consistent with the present results. Notably, these results differ from what is typically observed in repetitive activities, such as cycling (50), where significant changes in kinematic parameters are commonly reported and interpreted as indicators of local fatigue. However, the literature reports that in repetitive FT, local fatigue occurs only when the FT rate is externally imposed and sufficiently high (≥6 Hz). In contrast, when the tapping frequency is not imposed and remains below this critical threshold, fatigue is mainly of central origin. In the present study, the FT rate was approximately 5 taps per second. Participants were not required to adhere to a predetermined frequency; instead, they performed the task autonomously, with the only instruction to tap as fast as possible. This self-paced condition, coupled with a sub-threshold frequency, reinforces the notion that fatigue in this task is mainly driven by central rather than peripheral mechanisms (26–31).

However, a possible question remains regarding the mechanism underlying task termination, even if there are no signs of local fatigue. More specifically, it is stated that repetitive FT elicits a “central fatigue,” manifested in the primary motor cortex as a progressive decrease in surrounding inhibition on antagonist muscles (32). However, this situation could be effectively counteracted by the abovementioned strengthening of intracortical inhibition induced by FV. At the same time, prolonged contractions lead to the accumulation of catabolites at the muscle level, which increasingly stimulate ergoreceptors over time. These receptors are known to inhibit the activity of mechanoreceptors (51) and may, in turn, reduce the sensitivity of the muscle spindle, leading to notable disruptions of proprioception (52). These combined mechanisms may contribute to task termination; however, this remains a hypothesis in the absence of physiological confirmation. In conclusion, while the current study shows promising effects of PT administered via FV on coordinative tasks such as FT, the underlying physiological explanations should be regarded as speculative, and future studies incorporating direct neural measurements are therefore warranted to clarify these mechanisms.

## 6 Conclusion

This study highlights the potential role of PT using localized mechanical vibration in enhancing performance during a repetitive FT task. Significant improvements were observed immediately after the treatment ended and continued to increase over the next 4 weeks, whereas relatively small gains in the control group were attributed to a training effect over the same period. No modifications in movement kinematics were observed, supporting the hypothesis of a predominant involvement of CNS mechanisms, particularly those related to fatigue resistance.

However, it is important to note that these inferences are based solely on behavioral and kinematic data, without direct neurophysiological evidence. While FV has been described in the literature as a powerful stimulus capable of modulating motor commands, in the present study, its effect appears to extend to coordinative performance involving fine and precise finger movements. Notably, these effects occurred at an autonomous tapping rate, where local fatigue is negligible. Future research is needed to investigate the FV effect at tapping rates that are sufficient to induce local fatigue (≥6 Hz). Although further investigation is warranted, especially in clinical settings, the findings provide preliminary insight into non-invasive strategies that may support motor performance. Potential implications for quality of life and functional autonomy in motor rehabilitation contexts should be explored in future studies.

## Data Availability

The raw data supporting the conclusions of this article will be made available by the authors, without undue reservation.
